# 

**Published:** 1908-09-26

**Authors:** 


CONTENTS.

| | PAGE |
|---|---|
| **Lunatics at Large** | 669 |
| **A Question of Clothing** | 670 |
| **Annotations—** | |
| Charitable Swindles—The Cholera Epidemic—Indecent Advertisements | 671 |
| **Medical Opinion and Movement** | 672 |
| **Hospital Clinics—** | |
| Cancer of the Biliary Ducts | 675 |
| **Special Article—** | |
| Cholangitis without Jaundice | 676 |
| **Medicine—** | |
| Oxaluria—I. | 679 |
| Enlargements of the Spleen—VIII. | 680 |
| **Surgery—** | |
| Some Remarks on Hernia | 681 |
| Rectal Drainage of Pelvic Appendicular Abscesses | 682 |
| **The Royal Army Medical Corps Section—** | |
| The Territorial Medical Schools of Instruction | 683 |
| **The General Practitioner's Column—** | |
| Points in Prescribing | 684 |
| **Dental Department—** | |
| Dental Prophylaxis | 685 |
| **Ophthalmology—** | |
| Corneal Ulcerations | 686 |
| **Public Health and Hygiene—** | |
| The Evolution of a Civic Medical Service | 687 |
| **Hospital Administration—** | |
| Some Modern Continental Hospitals | 689 |
| **A Hint for Hospital Secretaries** | 690 |
| **New Appliances and Things Medical** | 690 |
| **News and Coming Events** | 691 |
| **Nursing Administration—** | |
| The Domestic Staff—V. | 693 |
| **Editor's Letter-Box** | 694 |
| **The Graduates' Medical Schools** | 694 |

